# Electricity Consumption Estimation of the Polymer Material Injection-Molding Manufacturing Process: Empirical Model and Application

**DOI:** 10.3390/ma11091740

**Published:** 2018-09-16

**Authors:** Ana Elduque, Daniel Elduque, Carmelo Pina, Isabel Clavería, Carlos Javierre

**Affiliations:** 1 BSH Electrodomésticos España S. A., Avda. de la Industria, 49, 50016 Zaragoza, Spain; anaelduque@gmail.com (A.E.); carmelo.pina@bshg.com (C.P.); 2 i+, Department of Mechanical Engineering, EINA, University of Zaragoza, C/María de Luna, 3, 50018 Zaragoza, Spain; iclaver@unizar.es (I.C.); carlos.javierre@unizar.es (C.J.)

**Keywords:** polymer material, injection-molding, manufacturing processes, electricity consumption, environmental impact, empirical model

## Abstract

Polymer injection-molding is one of the most used manufacturing processes for the production of plastic products. Its electricity consumption highly influences its cost as well as its environmental impact. Reducing these factors is one of the challenges that material science and production engineering face today. However, there is currently a lack of data regarding electricity consumption values for injection-molding, which leads to significant errors due to the inherent high variability of injection-molding and its configurations. In this paper, an empirical model is proposed to better estimate the electricity consumption and the environmental impact of the injection-molding process. This empirical model was created after measuring the electricity consumption of a wide range of parts. It provides a method to estimate both electricity consumption and environmental impact, taking into account characteristics of both the molded parts and the molding machine. A case study of an induction cooktop housing is presented, showing adequate accuracy of the empirical model and the importance of proper machine selection to reduce cost, electricity consumption, and environmental impact.

## 1. Introduction

Climate change is forcing companies to perform risk management as well as look for opportunities to reduce the environmental impact of their operations [1,2].

The concern regarding the achievement of sustainable development is patent in the literature. Strategies to achieve a cleaner industrial sector have been discussed using, for example, analytical tools that help in the decision-making of an industrial process [3] or linking lean manufacturing practices with the lifecycle assessment methodology in order to reduce the environmental impact [4].

Polymer injection-molding is a standard manufacturing process that is typically characterized by high production volumes [5]. Electricity is required during several steps of this process, from the movements of the machine that allow the closure of the mold, filling the cavity, holding and ejecting of the part, to the plasticizing phase of the polymer and the needs of cooling for both the machine and its parts. In order to carry out these steps of the cycle, injection-molding machines are composed of two different units: the injection unit and the clamping unit. The injection unit is responsible for heating the polymer up to the injection temperature by rotating the screw and using electric resistors. The clamping unit works as a press supplying a clamping force that allows the closure of the mold during filling. To avoid defects in the parts due to partial mold opening, this clamping force should be higher than the one exerted by the injection pressure during the filling phase of the molding process. A wide range of injection machines are used in the industry, with their clamping force being the main distinguishing characteristic. According to the way the movements of the machines are powered, we can divide them into three groups: hydraulic (pumps), hybrid (hydraulic with another mechanism to help the hydraulic system), and electric (servomotors), with the last one being the most well recognized for its more efficient technology.

As injection-molding is an important manufacturing process, analyzing its environmental impact—and identifying ways to reduce it—is of interest to achieve a more sustainable development in the industrial sector.

As discussed in earlier work by the authors, the electricity consumption of the injection-molding process is the main factor that generates environmental impact [6,7]. Due to this, achieving savings in the electricity consumption will not only directly reduce the environmental impact but will also reduce the economic cost.

Therefore, injection-molding producers should have the goal of possessing an in-depth knowledge of their machines in order to ably optimize their consumption, while obtaining these two clearly important benefits.

Life Cycle Assessment (LCA) is a methodology that allows researchers to quantify the environmental impact of a product, process, or service. LCA studies have been applied to a wide range of products in most work fields [8,9,10,11,12,13,14]. Increasing knowledge about the injection-molding process will also lead to an improvement in the LCA field because databases such as EcoInvent, which are used in the Life Cycle Inventory (LCI) phase, are key to the results obtained in LCA studies.

EcoInvent LCI dataset for the injection-molding process is created by calculating the average of three processes—injection-molding of poly(vinyl chloride) (PVC), polypropylene (PP), and polyethylene terephthalate (PET)—and considering an average electricity consumption (1.47 kWh/kg) as well as an average consumption of water, lubricating oils, chemicals, fillers, solvents, packaging materials, natural gas for the factory, generated waste, etc. [15]. However, as previously explained, the highest contribution to the environmental impact is caused by the electricity consumption [6,7].

Not many studies with experimental data of injection-molding and its electricity consumption can be found in the literature. Mianehrow and Abbasian published an interesting study of monitoring energy [16], which analyzed how different factors, such as machine technology or process-related parameters, affect electricity consumption, with the cycle time and throughput (kg processed per hour) being the most important factors. More studies have been published in recent years regarding the environmental impact of the injection-molding process. Thiriez reviewed the complete process, including the compounding of the raw material. In this research, it was indicated that the type of injection-molding machine has a significant impact on the electricity consumption of the process, as will also be seen in this paper [17]. Other authors focused their research on the environmental performance of biodegradable polymers by studying its manufacturing process. They highlighted the necessity of following a holistic approach, considering, at the same time, the influence of the process when designing a part [18,19]. Studies concerning the estimation of electricity consumption of the injection-molding process have been carried out using a theoretical approach instead of experimental, such as the one used in this paper [20,21]. Spiering et al. remarked the importance of deeply analyzing the life cycle inventories of manufacturing processes [22].

In this research work, results from experimental measurements have been analyzed in order to define an empirical model that allows the estimation of electricity consumption of a specific injection-molding process depending on several parameters. Through this empirical model, a more precise value can be obtained to further assess the environmental impact and the costs related to this manufacturing process.

Moreover, the environmental impact of the manufacturing process of the parts used to build the model will be calculated in Section 3.2 using these electricity consumption values as the key for calculation.

A case study will also be carried out in Section 3.3, comparing several scenarios for the manufacturing of a plastic component. Employing this case study, the empirical model can be validated for values outside the ones originally used to create the model, showing the applicability of this model and the implemented methodology.

## 2. Materials and Methods

As the purpose of this paper was to obtain an empirical model, a spreadsheet was used to analyze and represent all the experimental data. Many tendencies were observed and studied. In the following subsections, the steps that were followed and the parameters selected to build the empirical model are explained in detail.

### 2.1. Experimental Data Analysis

As indicated by the authors in Reference [23], several tendencies could be analyzed from the 36 experimental measurements that were carried out. Twelve different injection-molding machines were analyzed in these measurements—from an all-electric injection machine with a clamping force of 850 kN, to a large injection-molding machine (80,000 kN clamping force).

One of the most significant tendencies was that the higher the throughput (kg/h) of the process, the lower was the SEC (specific energy consumption, kWh/kg) obtained for the injected part. On the other hand, each injection-molding machine showed slightly different tendencies as their technology and efficiency were not the same.

After analyzing all the experimental measurements and considering the high variability depending on the characteristics of the parts and the machine, it was proposed to develop an empirical model in order to predict SEC of other components when energy consumption measurement is not possible.

Analyzing the studied tendencies for the experimental measurements, the following parameters were selected to build this empirical model:

- Percentage of the machine’s utilization (%): relation between part injected volume and maximum volume that can be injected in one shot
- Machine’s efficiency (%)
- Throughput (kg/h)
- Polymer material (Specific heat [kJ/kg∙K]∙ΔT)

With these four parameters, the following factors were considered: the influence of injection-molding machine (i.e., its technology), how well the machine and the process are optimized concerning the injected part, and the properties of the polymer material. All these factors were determined as relevant in electricity consumption after analyzing all the experimental measurements.

A total of 36 measurements were used using different thermoplastics, such as high-density polyethylene, polypropylene, polycarbonate, polyamide with several percentages of fillers, etc. Further details regarding these data (parts injected, measurement equipment to obtain consumption values, etc.) are further detailed in an earlier published work by the authors [23].

In the following section, the building of the empirical model and the obtained results will be explained.

### 2.2. Empirical Model

**Figure 1:**
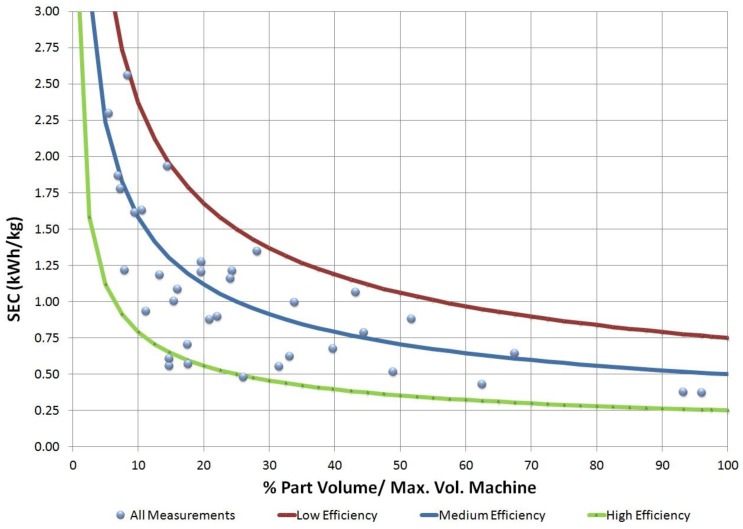
Empirical model.

After analyzing all the measurements, an empirical model was developed to obtain a part SEC, depending on the percentage of the machine’s utilization as well as its material and efficiency. In order to do that, several steps were conducted to develop an empirical model. First, the electricity consumption (SEC) was modeled considering the percentage of utilization and the machine’s efficiency. Figure 1 shows all experimental measurements in blue dots. The low, medium, and high-efficiency lines show the limits of the SEC depending on the efficiency of the injection machine.

Table 1 shows the electricity consumption values obtained by the model for the three cases shown in Figure 1: low, medium, and high-efficiency machines.

Values of Table 1 were obtained from Equation (1), which was determined by a regression analysis of Figure 1:

SEC (KWh/kg) = (7.5 − 5× E/100) × (η)^0.5^ (1)

where E is the efficiency of the injection machine, and η is the percentage of utilization of the injection-molding machine, which is defined as Equation (2):

η = w×100/ρ× V_max_ (2)

where w is the part’s weight in grams, ρ is the density of the polymer material (g/cm^3^), and V_max_ is the maximum value that the injection-molding machine is able to inject (cm^3^)—a value that can be obtained from the machine datasheet. In addition, a value for 0% of utilization has been included in Table 1 as a reference because the SEC of an obtained part cannot be infinite.

In this second step, two correction factors were introduced in order to modify the previously shown efficiency and increase the accuracy of the model. The first correction factor was related to the throughput of the process. A higher throughput would lead to a more optimized process (higher value of efficiency and, therefore, lower electricity consumption). An empirical value was calculated to estimate the average throughput depending on the clamping force (F_c_) of the injection-molding machine using a linear regression with a correlation factor of 0.93 (Equation (3)):

Average Throughput (kg/h) = 0.0052× F_c_ (kN) (3)

This way, the Correction Factor of the Throughput (CFT) would be defined as Equation (4), with t_c_ being the cycle time in seconds:

CFT = (w×3.6/t_c_)/0.0052× F_c_ (kN) (4)

Equation (5) defines the second correction for the modified efficiency, which adds the influence of the material, i.e., the Correction Factor of the Polymer (CFP):

CFP = (c_e_× (T_i_ − T_a_))/350.255 (5)

This CFP was calculated using the specific heat of the polymer material (c_e_) and the difference between injection temperature (T_i_) and ambient temperature (T_a_). The 350.255 coefficient was an experimental value obtained as an average of the factor c_e_ × (T_i_ − T_a_) in the measurements.

Therefore, the modified machine’s efficiency, E’, which replaces E in Equation (1), was defined in the model with Equation (6), where the influence of the throughput was higher than that of the polymer material:

E’ = E × (CFT)^0.15^/(CFP)^0.1^ (6)

The considered efficiency for each injection-molding machine was selected considering its technology and manufacturing year, as shown in Table 2. The 0.15 and 0.1 exponents of Equation (6) were obtained by minimizing the error of the empirical model versus the 36 experimental measurements.

Taking all these factors into account, the final equation of the empirical model is shown in Equation (7):

SEC = (7.5 − (5× (E/100) × (((w×3.6/t_c_)/(0.0052× F_c_))^0.15^/(c_e_× (T_i_ − T_a_)/350.255)^0.1^))) × (w×100/(ρ× V_max_))^0.5^ (7)

### 2.3. Environmental Impact Assessment

Using the methodology proposed in [7], the environmental impact of the process can be very precisely calculated with the value of SEC. The functional unit is the processing of 1 kg of polymer through an injection machine.

When applying this methodology, a customized dataset was created to assess the environmental impact of the process. This customized dataset was built analyzing the original data of EcoInvent, which creates it as an average of the processing values of three polymers: PVC, PP, and PET [15]. After analyzing these values, a customized dataset was proposed using the values for the most conventional plastic—PP—and excluding inputs not directly related to the processing, such as packaging materials or fuels for factory heating. In this way, elements such as lubricant and water or the infrastructure of the factory were considered as well as the plastic waste generated and the electricity consumption of the process (which in this case will be replaced by measured or modeled values for each part to obtain a more accurate result). The typical European electrical mix was used [24]. ReCiPe EndPoint (H/A) was used as a calculation methodology as it considers several impact categories and normalizes and weighs all the results to obtain only one value in millipoints (mPt), which allows an easier interpretation of the result [25]. In addition, the Global Warming Potential category (equivalent kilograms of CO_2_) IPCC 2013 100a GWP was calculated due to its high social relevance [26].

## 3. Results and Discussion

After obtaining the empirical model, it was first compared against the experimental measurements to analyze how this model behaves and then against a case study of a PP injection-molded component of an induction cooktop that was not previously used to obtain the empirical model.

### 3.1. Empirical Model

**Figure 2:**
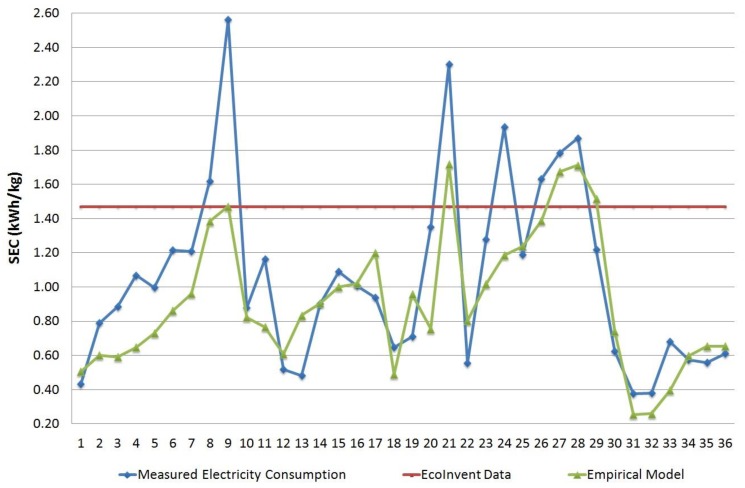
SEC comparison of the model’s results, measured values, and the EcoInvent data.

Figure 2 displays the empirical model SEC results and compares them with measurement data and with EcoInvent value for the electricity consumption of the injection-molding process for 1 kg of thermoplastic. All the characteristics of the 36 combinations are shown in Table S1 (Supplementary Materials).

An average absolute error of 22.5% was obtained for the empirical model, which was lower than the 86.8% absolute error obtained using EcoInvent value for this set of measured parts.

Part #9 registered the maximum error in this empiric model (−37%). This part was injected in one of the largest machines (C, 30,000 kN), had a high thickness, and required a high cycle time for its processing, which led to very low kg/h and η values. As can be seen in Figure 1, for low values of utilization, the model’s uncertainty increased as the electricity consumption result was much more sensitive to changes in the modified machine’s efficiency (E’).

### 3.2. Environmental Impact

**Figure 3:**
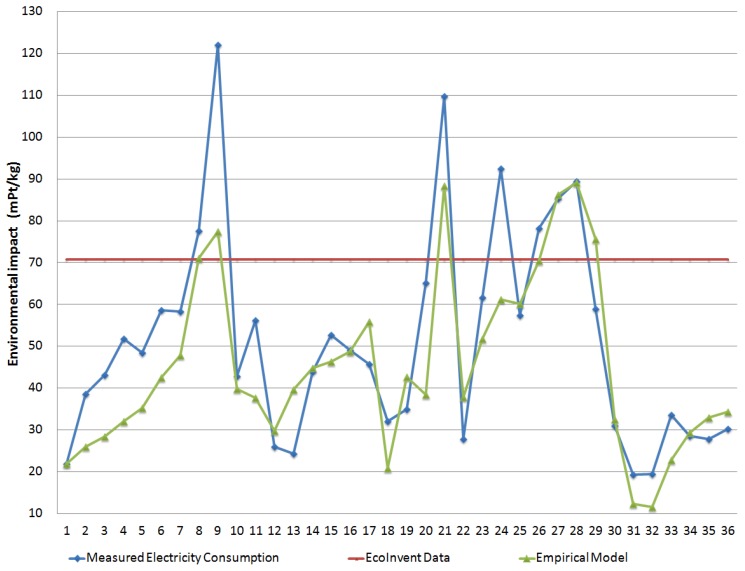
Comparison of modeled, measured, and EcoInvent data for the environmental impact assessment (ReCiPe).

In Figure 3, the comparison of the environmental impact results is performed, considering the measured, modeled, and EcoInvent value of electricity consumption for the injection-molding process of 1 kg of polymer. Results are indicated in mPt per kg using the ReCiPe methodology [24].

Figure 3 shows the same tendency as the previous graphics because electricity consumption is the most significant factor in the environmental impact results of this manufacturing process. Part#9 was also the one that registered the higher difference in the ReCiPe results between the empirical model and the measured values of SEC.

The average for the environmental impact results with ReCiPe methodology using the empirical model was 45.1 mPt/kg (51.2 mPt/kg using measured SEC values), while the value using EcoInvent SEC (constant value of 1.47 kWh/kg) was 70.7 mPt/kg.

For this set of parts, an average absolute error of 21.5% was obtained with the empirical model for ReCiPe results, which was lower than the 81.7% absolute error obtained when using EcoInvent values.

**Figure 4:**
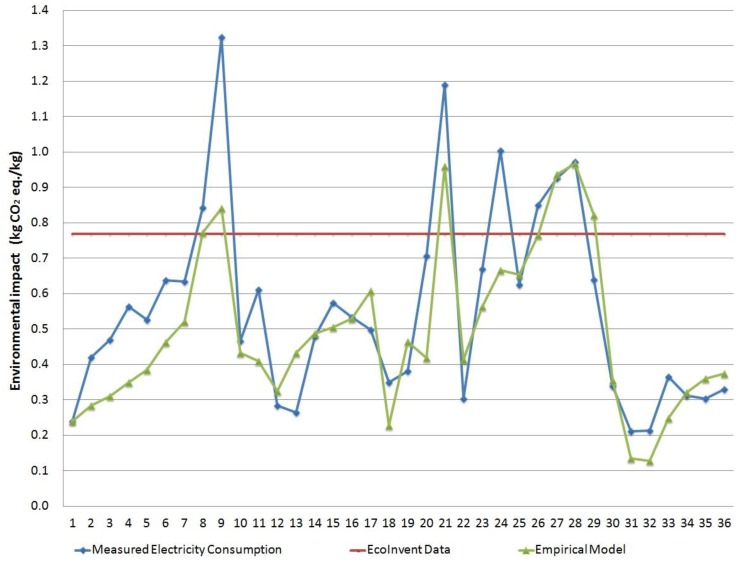
Comparison of modeled, measured, and EcoInvent data for the environmental impact assessment (kg equivalents of CO_2_).

Results obtained with Carbon Footprint methodology [26] are shown in Figure 4. Again, they followed the same tendencies as the previous ones due to the importance of electricity consumption in the results. The average results with this methodology was 0.56 equivalent kilograms of CO_2_ using measured SEC values, 0.49 for the empirical model, and 0.77 using EcoInvent electricity value. The average absolute error for the empirical model was 21.2%, which was much more accurate than the 80.1% achieved with the EcoInvent dataset.

### 3.3. Case Study: Induction Cooktop Housing

**Figure 5:**
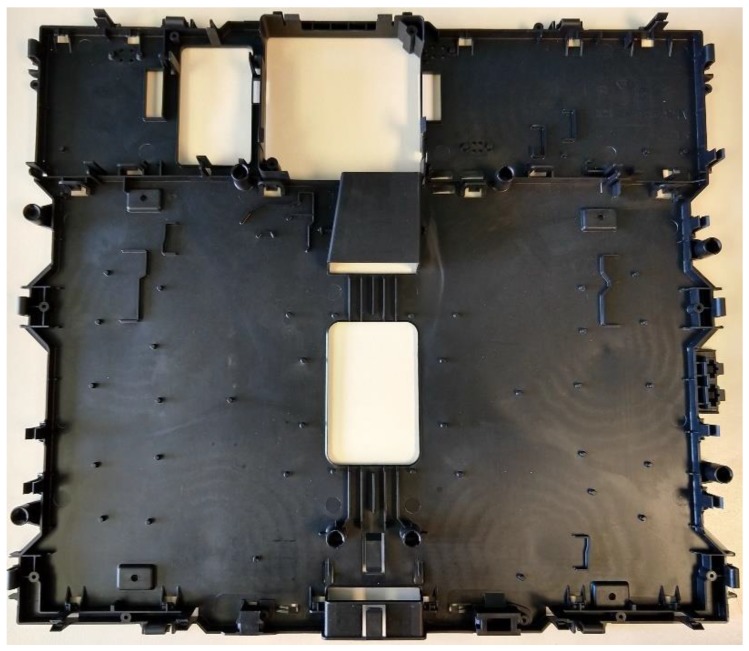
Induction cooktop—internal housing.

In the following section, a case study for an induction-hob-injected plastic component (Figure 5) is presented. This element is used as a housing for the electronic boards of an induction cooktop, and their general dimensions are 534 × 472 mm^2^. As more than a million units of this component are produced each year, assessing its consumption and its environmental impact is of interest.

In the case under investigation here, two polypropylene materials were studied: virgin polypropylene versus an equivalent recycled one [27]. The components were injected using two different injection-molding machines; therefore, four different scenarios were analyzed. This fact allowed the behavior, particularly, the errors of the empirical model, to be checked by measuring new data that had not been previously used in the process of model adjustment, as shown in Section 2.2. The two injection-molding machines used will be addressed from now on as “M” and “N” machines.

Table 3 shows the main data for the four scenarios combining the two materials and the two injection-molding machines. “M” injection-molding machine had 8000 kN of clamping force and a toggle clamp system, whereas “N” injection-molding machine had 5500 kN of clamping force. Both machines were manufactured in 2015. Considering the assigned values in Table 2 and the characteristics of “M” and “N” machines, values of E of 85 and 75, respectively, were assigned to compare the characteristics of these machines with the ones that have already been characterized and were used to develop the empirical model. A higher efficiency was assigned to the “M” machine as it had a toggle clamp system that allowed savings in the machine movements.

Table 3 shows the maximum volume that could be plasticized in the injection unit of each machine. The capacity of machine “N” was much more adjusted to the volume of the component than the machine “M” (1500 vs. 3240 cm^3^). Cycle time, gross injected weight, specific heat of the polymers, and the differences of injection and ambient temperature were also included.

Ultimately, the different SEC of measurements can be compared in Table 3 with those obtained with the empirical model and with EcoInvent data.

Machine “M” registered higher measured SEC values than that for “N”. The empirical model correctly predicted this point as machine “N” was better adjusted for the processing of this part.

The model estimation was, as expected, more accurate than the EcoInvent value with an average absolute deviation of 3.9% compared to the 114% of EcoInvent’s deviation for these four scenarios. Depending on the machine used, the recycled material obtained a higher or lower value than the virgin material as there were minor differences between both materials. It can also be observed that the cycle times for the recycled materials were higher. This increase was required to meet dimensional requirements as both materials have slightly different shrinkage rates.

In Table 4, the environmental impact results per part are represented for the four proposed scenarios using the previously explained methodology and the measured data of electricity consumption. Results are displayed in mPt ReCiPe and IPCC 2013. Although there were no significant differences in the environmental impact of processing virgin or recycled material, under a holistic approach and taking into account the complete life cycle, the use of recycled material would help to reduce the environmental impact.

## 4. Conclusions

In this paper, an empirical model was proposed to achieve better estimations of the electricity consumption of the injection-molding process. Databases such as EcoInvent provide an average electricity consumption value (1.47 kWh/kg) for the injection-molding process; however, considering this value as a constant for every injected molded part leads to significant errors as the variability in these processes is very high. Experimental measurements revealed that SEC highly depended on the polymer material, the injection-molding machine used, and the parameters of the process. Our empirical model obtained an average SEC absolute error of 22.5%, which was much lower than the 86.8% obtained when using EcoInvent data.

These empirical modeled SEC values were used to calculate the environmental impact of the parts as electricity consumption is the main factor for the injection-molding process. It was observed that the influence of the injection-molding machine and the parameters of the process were higher than the influence of the polymer material. This observation is valid for assessing the energy consumption and the environmental impact.

A case study using an induction cooktop PP housing showed that the empirical model provided good estimations for the SEC values. This validated the model as it was checked analyzing measurements that had not been previously used to develop it.

For the component, two PP materials (virgin and recycled) were analyzed and they were processed in two different injection-molding machines. Components processed in machine “M” had higher cycle times and a lower percentage of utilization as it had a higher capacity than the “N” machine. Both factors led to an increase in the SEC, showing the importance of proper machine selection.

This study has demonstrated that each injection-molding machine has a distinctive electricity consumption profile due to its efficiency. Therefore, plastic processing companies should carry out a benchmark of their injection machines in order to correctly characterize their efficiency and be able to enhance the machine selection process.

Although the developed empirical model provided appropriate results, future lines of research will be focused on increasing the number of experimental measurements in other injection-molding machines with different clamping forces and technologies in order to improve the empirical model and, therefore, the SEC and environmental impact calculations.

## Figures and Tables

**Table 1:** Estimated electricity consumption in kWh/kg for different values of machine efficiency and % of utilization.

% of Utilization	Low-Efficiency SEC	Medium-Efficiency SEC	High-Efficiency SEC
0	6.000	5.000	4.000
10	2.372	1.581	0.791
20	1.677	1.118	0.559
30	1.369	0.913	0.456
40	1.186	0.791	0.395
50	1.061	0.707	0.354
60	0.968	0.645	0.323
70	0.896	0.598	0.299
80	0.839	0.559	0.280
90	0.791	0.527	0.264
100	0.750	0.500	0.250

**Table 2:** Machine efficiency values for the empirical model, E.

Injection-Molding Machine	Clamping Force (kN)	Manufacturing Year	Efficiency (E)
A	80,000	2005	70
B	52,000	2005	70
C	30,000	2000	65
D	20,000	2010	75
E	16,500	2010	75
F	12,000	1999	65
G	10,000	2008	70
H	7500	2005	70
I	4000	1996	60
J	2000	1999	65
K	1250	1999	65
L	850 (All-electric)	2002	100

**Table 3:** Case study data summary.

Scenario	#1	#2	#3	#4
Polymer Material	Virgin PP	Recycled PP	Virgin PP	Recycled PP
Injection-Molding Machine	M	M	N	N
Fc (kN)	8000 (with toggle clamp system)	8000 (with toggle clamp system)	5500	5500
Manufacturing Date	2015	2015	2015	2015
E	85	85	75	75
V_max_ (cm^3^)	3240	3240	1500	1500
Cycle time (s)	51.41	60.81	38	39
w (g)	595	603.2	606	615
ρ (g/cm^3^)	1.23	1.25	1.23	1.25
c_e_ (KJ/kg. K)	1.5	1.5	1.5	1.5
T_i_ − T_a_ (K)	228	228	228	228
Measured SEC (kWh/kg)	0.838	0.785	0.59	0.601
Modeled SEC (kWh/kg)	0.835	0.861	0.578	0.580
Model Abs. Error	0.4%	9.7%	2.0%	3.5%
EcoInvent Abs. Error	75.4%	87.3%	149.2%	144.6%

**Table 4:** Scenarios Assessment: Environmental impact of processing a plastic part.

Environmental Impact of Part Processing	#1	#2	#3	#4
mPt ReCiPe/Part	24.41	23.24	17.80	18.38
kg eq. CO_2_/Part	0.266	0.253	0.194	0.200

## Supplementary Materials

The following are available online at http://www.mdpi.com/1996-1944/11/9/1740/s1, Table S1: Measured Parts Characteristics and Injection-Molding Machine.
