# Correction: Elduque, A., et al. Electricity Consumption Estimation of the Polymer Material Injection-Molding Manufacturing Process: Empirical Model and Application

**DOI:** 10.3390/ma13112548

**Published:** 2020-06-03

**Authors:** Ana Elduque, Daniel Elduque, Carmelo Pina, Isabel Clavería, Carlos Javierre

**Affiliations:** 1 BSH Electrodomésticos España S. A., Avda. de la Industria, 49, 50016 Zaragoza, Spain; anaelduque@gmail.com (A.E.); carmelo.pina@bshg.com (C.P.); 2 i+, Department of Mechanical Engineering, EINA, University of Zaragoza, C/María de Luna, 3, 50018 Zaragoza, Spain; iclaver@unizar.es (I.C.); carlos.javierre@unizar.es (C.J.)

During the formatting phase of this article [1], the authors did not detect a typographical error in the equations to calculate the SEC value; they would therefore like to make the following correction in both Equations (1) and (7). There was a minus symbol missing in the super index of the η value (percentage of utilization of the injection-molding machine).

Equation (1) was wrongly formatted as:

SEC (KWh/kg) = (7.5 – 5 × E/100) × (η)^0.5^ (1)

Moreover, it must be corrected as the following:

SEC (KWh/kg) = (7.5 – 5 × E/100) × (η)^−0.5^ (1)

Equation (7) was wrongly formatted as:

SEC = (7.5 − (5 × (E/100) × (((w × 3.6/t_c_)/(0.0052 × F_c_))^0.15^/(c_e_× (T_i_ − T_a_)/350.255)^0.1^))) × (w × 100/(⍴ × V_max_))^0.5^ (7)

It must be corrected as the following:

SEC = (7.5 − (5 × (E/100) × (((w × 3.6/t_c_)/(0.0052 × F_c_))^0.15^/(c_e_ × (T_i_ − T_a_)/350.255)^0.1^))) × (w × 100/(⍴ × V_max_))^−0.5^ (7)

The authors would also like to take the opportunity to apologize for any inconvenience that this typographical error may have caused in the calculations done by the readers. All the data, calculations and results are correct, but both equations were missing, in the final version, the minus symbol in the superscript, due to a formatting error.
