# Supplementary material for: Electricity Consumption Estimation of the Polymer Material Injection-Molding Manufacturing Process: Empirical Model and Application
Source: Materials (Basel). 2018 Sep 16;11(9):1740. doi: 10.3390/ma11091740 (PMC6164802; doi:10.3390/ma11091740)
Supplement: Supplementary file 1 [file materials-11-01740-s001.pdf]

materials

Supplementary

Electricity Consumption Estimation of the Polymer Material Injection-Molding Manufacturing Process: Empirical Model and Application

Ana Elduque, Daniel Elduque, Carmelo Pina, Isabel Clavería and Carlos Javierre

Table S1. Measured Parts Characteristics and Injection-Molding Machine, [23].

Part	Polymer Material	Injection-Molding Machine	Weight (g)	Cycle time (s)	Throughput (kg/h)
1	HDPE	A	71800	216.00	1196.67
2	HDPE	B	30300	147.00	742.04
3	HDPE	C	10500	175.00	216.00
4	HDPE	C	8700	194.00	161.44
5	PP	C	7258	140.20	186.37
6	PP+EDPM+PE+10T	C	4695	118.00	143.24
7	PP+EDPM+10T	C	3773	106.00	128.14
8	PP+EDPM+15T	C	1802	77.60	83.60
9	PP+EDPM+20T	C	1589	91.00	62.86
10	PC	D	646	22.00	105.71
11	PC	E	745	33.70	79.58
12	HDPE	F	2778	139.00	71.95
13	PP	F	1560	70.00	80.23
14	HDPE	F	1253	81.00	55.69
15	PC	G	495	24.40	73.03
16	PMMA	G	489	29.05	60.60
17	SAN	G	383	24.55	56.16
18	PP	H	3407	100.00	122.65
19	HDPE	H	836	40.00	75.24
20	HDPE	H	1336	162.00	29.69
21	HDPE	H	260	42.90	21.82
22	POM	I	310.75	37.60	29.75
23	PP	I	288	84.00	12.34
24	PA+50% LF	I	128.58	48.00	9.64
25	PA	I	161.24	29.20	19.88
26	HDPE	I	154.12	47.68	11.64
27	HDPE	I	106.00	40.30	9.47
28	HDPE	I	100.50	44.40	8.15
29	ABS	J	39.58	22.00	6.48
30	PA	K	67.66	14.00	17.40
31	PA+30% GF	L	68.00	37.20	6.58
32	PP (100% recycled)	L	100.48	45.00	8.04
33	ABS	L	37.00	53.00	2.51
34	PA	L	16.08	11.80	4.91
35	PP	L	14.36	12.50	4.14
36	HDPE	L	15.00	15.00	3.60

© 2018 by the authors. Submitted for possible open access publication under the terms and conditions of the Creative Commons Attribution (CC BY) license (http://creativecommons.org/licenses/by/4.0/).
